# Editorial: Novel approaches to prevention, diagnosis, and treatment of bacterial and viral infections of clinical relevance

**DOI:** 10.3389/fmicb.2023.1192435

**Published:** 2023-04-13

**Authors:** Leon G. Leanse, Claudia Maria Trombetta, George W. Carnell

**Affiliations:** ^1^Health and Sport Sciences Hub, University of Gibraltar, Europa Point Campus, Gibraltar, Gibraltar; ^2^Wellman Center for Photomedicine, Massachusetts General Hospital, Harvard Medical School, Boston, MA, United States; ^3^Department of Molecular and Developmental Medicine, University of Siena, Siena, Italy; ^4^Department of Veterinary Medicine, University of Cambridge, Cambridge, United Kingdom

**Keywords:** bacterial diagnosis, viral diagnosis, infection treatment, vaccination, novel application

In the era of antimicrobial resistance, infectious diseases are ranked among the most serious global health threats. This concern continues to be perpetuated by the current COVID-19 pandemic which has reinvented the scientific, cultural, and sociological paradigm that drives daily life, illuminating the fact that the ongoing emergence and re-emergence of pathogenic microorganisms place a burden on public health. Considering that the ongoing emergence and re-emergence of pathogenic microorganisms place a burden on public health, therapeutic antimicrobials and vaccines are essential approaches to control infections and prevent pandemic occurrences. However, as new pathogens emerge, therapeutic and vaccine approaches are not always readily available. This is further complicated by pathogens that express variable antigenicity, rendering effective vaccine development difficult. The treatment of pathogens is similarly convoluted in that many infection syndromes present with similar constellations of symptoms, which can obscure the selection of appropriate treatment methods. The current gold-standard methods for diagnosis of bacterial and viral infections often take hours or days to give accurate results that would permit healthcare providers to initiate appropriate treatment, potentially increasing the chances of adverse effects. More worryingly, even with an accurate diagnosis, the ubiquity of pathogens that are resisting conventional therapeutics coupled with those complex pathogens that have no effective or gold-standard treatment within the clinical pipeline (e.g., SARS-CoV-2) makes controlling infectious diseases more difficult. Thus, the development of novel prevention, diagnosis, and treatment methods that effectively control infectious diseases is essential for integration into the clinical pipeline.

Our special issue entitled ‘Novel approaches for diagnosis, prevention, and treatment of bacterial and viral infections of clinical relevance' tackles these very concerns listed above. With a total of 13 articles published within our special issue, many of the research articles are one step closer to adjusting the clinical pipeline to improve current prevention, diagnostic, and treatment platforms against a myriad of bacterial and viral infections of clinical importance.

A study by Rua et al. attempted to improve the diagnosis of bacterial and fungal infections. They utilized Positron emission tomography (PET), which is a non-invasive imaging technique to identify the sites of infection. The authors identified that 2-deoxy-2[^18^F]fluoro-D-glucose: [^18^F]FDG, a commonly used radiotracer used in PET, demonstrated uptake at regions of inflammation. However, it does not discriminate between sterile and infectious inflammation. Thus, they evaluated two infection-specific radiotracers 2-deoxy-2-[^18^F]-fluoro-D-sorbitol ([^18^F]FDS) and 2-[^18^F]F-ρ-aminobenzoic acid ([^18^F]FPABA using 19 bacterial and fungal (yeast) species *in vitro* or within a mouse model with myositis infection. They found that non-lactose fermenters were unable to take up [^18^F]FDS *in vitro*. Impressively, [^18^F]FDS could be visualized in *Escherichia coli in vivo*. In addition, it could discriminate between yeasts that have differential sorbitol assimilation capabilities. Conversely, uptake of [^18^F]FPABA was possible in all bacterial and fungal species. Additionally, it could adequately discriminate between inflammation and infection in the myositis model. The authors concluded that the PET approach using [^18^F]FPABA may offer an approach to assist in the diagnosis of bacterial and fungal infections.

A complimentary manuscript by Ting et al. touched upon the difficulties associated with the diagnosis of infectious keratitis (IK). They explained how IK is the 5th leading cause of blindness, and how timely diagnosis is essential to mitigate any adverse reactions. They stated that this is not always possible due to clinical delays complicated by a lack of education and the use of medicinal eye drops. A low culture yield, the long turnaround time for culture results, undifferentiated clinical phenotypes, or polymicrobial infection has been shown to further complicate timely diagnosis. They described potential solutions targeted at improving the timeliness of diagnosis. For example, the use of digital innovations such as artificial intelligence-directed imaging techniques to identify an ocular infectious burden. They discussed the use of metagenomic next-generation sequencing (NGS) methods, initially touching upon the use of polymerase chain reaction (PCR) as a strategy to diagnose infectious diseases. With the advancement in NGS technologies, they provided evidence that NGS can identify IK even when the sample in the culture is negative or if the patient was pre-treated with antimicrobials. They concluded that further advancement of digital and NGS methods may change the current IK diagnostic framework. They remain, however, cognizant of the feasibility related to implementing these methods based on accessibility and cost effectiveness.

In another “diagnosis-driven” review, Raeisi et al. touched upon the use of rapid-format recombinant antibody-based methods for the diagnosis of *Clostridiodes difficile* as an effective way to provide a timely diagnosis to limit the rate of infection recurrence and improve the clinical outcome. They touched upon the traditional diagnostic method [i.e., detection of TcdA/TcdB toxins *via* enzyme-linked immunosorbent assay (ELISA)] given that studies demonstrated that their expression is restricted to pathogenic strains. Other methods of diagnosis include cell-culture cytotoxicity neutralization assay, and PCR. The authors suggested that the currently used methods suffer from drawbacks in the form of specificity/sensitivity issues such as long turnaround times, use of polyclonal (pAb) and monoclonal antibodies (mAb) for applications in immunofluorescence, and ELISA, and the methods are more advantageous relative to conventional antibodies given their accurate and reliable production. They are also beneficial as they can be fused with other proteins (i.e., detector enzymes) that increase the rate of reaction and assay sensitivity, thus improving on current gold-standard methods (e.g., ELISA). The authors concluded that recombinant antibodies (rAb) could be a “future trend” when designing highly sensitive diagnostic methods for *Clostridiodes* difficile infection.

Sohail et al. touched upon the role of infectious diseases on Diabetes Mellitus (DM) pathogenesis, specifically patients with DM who are at a high risk of hospitalization and/or death from infectious diseases, with inadequate control of blood glucose levels driving a myriad of infection syndromes. Thus, the authors proposed that a timely infection diagnosis coupled with appropriate glycemic control may be the key to limiting these adverse effects. They suggested that novel high-throughput blood-based immunoassays capable of detecting both infection and hyperglycemia are warranted. The authors presented the most common infections linked to DM and the disease pathogenesis and the use of immunoproteomics in an early infection diagnosis, concluding that the use of antibody screening should be a standard procedure with infection/diabetic screening.

In line with the above-mentioned studies, Wei et al. evaluated a novel rapid nucleic acid amplification method, chips for complicated detection (CCID) based on Loop-mediated isothermal amplification (LAMP), to diagnose elderly patients suffering from pneumonia. In their study on 81 patients, where 57 samples were isolated *via* sputum/airway secretion and 35 from bronchoalveolar lavage, they found the CCID results to be available in 50 min with a limit of detection of 500 copies/reaction. Additionally, they found the sensitivity of CCID to supersede conventional microbiological tests (CMTs), with the percentage of positive results in patients pre-treated with antibiotics for >3 days similarly higher in CCID vs. CMT (91.9% vs. 64.9%). The authors concluded that CCID may offer a rapid approach in the detection of pneumonia in older patients, with the added benefit of being highly sensitive even in patients treated with antibiotics.

In another study by Zhang et al., another novel method, targeted nanopore sequencing (TNPseq), to identify pathogens (and antimicrobial resistance (AMR) genes) causing lower respiratory tract infections (LRTIs) was applied. They recruited 146 patients with suspected LRTIs (median age: 61 years) and used traditional culture or TNPseq to identify pathogens and their associated AMR genes. In 91.1% (133/146) of patients, they identified at least one pathogen using TNPseq. Conversely, using traditional culturing, they only identified pathogens in 37/146 samples (25.3%), illustrating a higher sensitivity of TNPseq, relative to culture. Additionally, TNPseq detected more bacteria and mixed infections, compared to culture. Lastly, TNPseq was effective in detecting AMR genes, such as *bla*_TEM_. The authors concluded that TNPseq can efficiently detect pathogens early, thus, providing patients with accurate and timely treatment of LRTIs.

As the COVID-19 pandemic continues, novel diagnostic and treatment regimens continue to be developed such as a study by Pratelli et al. who developed a pooling strategy of real-time PCR (RT-PCR) to detect SARS-CoV-2. The authors postulated that the detection of SARS-CoV-2 within pooled samples would be a suitable and rapid approach for the mass monitoring of COVID-19 within a population. In their study, they used 17 patients who had previously tested positive for COVID-19 and 19 who were negative, using retested nasal swabs (NS) for their pooling strategy. For the test, one swab from a positive patient was mixed with 19 negative samples (20 NS in total). The pooling strategy detected SARS-CoV-2 to a similar extent to individual tests, demonstrating that even a low viral load is detectable. The authors concluded that this may be a suitable approach to routinely monitor SARS-CoV-2 within a population with low prevalence.

A study by Yu et al. sought to identify biomarkers that indicate liver injury as a result of chronic hepatitis B (CHB). Due to the incidences of hepatocellular carcinoma (HCC) that is associated with CHB, the inability to treat with a hepatectomy, which is the only treatment, increases the importance of identifying liver injury early. Given that expression of cytokines within the liver microenvironment has been shown to mediate liver damage, the authors investigated whether there is a correlation between five specific chemokines (CXCL8, CXCL9, CXCL10, CXCL11, and C-X-C-motif) to see if they may be used as predictors of liver injury. In their study, they recruited 28 healthy patients, 45 patients that suffer from CHB, and 20 patients that do not have HBV infection but have alanine aminotransferase (ALT) concentrations that are comparable to CHB patients. The authors concluded that CXCL8, 9, 10, and 11 can be used as biomarkers to forecast liver injury as a result of HBV infection.

Within the prevention component of our Research Topic, we have a study by Ren et al. that is concerned with the development of an influenza vaccine that confers heterologous protection with H5N1 and H9N2 subtypes. They constructed a replication-deficient recombinant influenza virus, WM01ma-HA(H5), that expresses a chimeric hemagglutinin (HA) protein from H5N1 and H9N2, with flanking neuraminidase (NA) sequence from A/Mink/Shandong/WM01/2014(H9N2)(WM01ma). The authors found this recombinant influenza strain to induce a significant immune response with complete immune protection (following intranasal inoculation), which was observed within a mouse model with H9N2 or H5N1 influenza infection. The authors hope their recombinant candidate vaccine will be effective in preventing future influenza pandemics.

A novel investigation by Xu et al. was performed that evaluated the efficacy mechanism of the Lianhua Qingwen capsule (LHQW), a traditional Chinese medicine, to promote immune function in a mouse model with influenza A infection. Specifically, they aimed to look at the role of intestinal microbiota in reducing pneumonia caused by influenza A infection. In infected mice, they detected influenza within the lungs; histologically evaluated tissue from the lungs and small intestine and biochemical inflammatory indices assessments were performed to assess the severity of the infection and potential amelioration. Additionally, they used 16s rRNA analyses to look at the intestinal microbiota, and network pharmacology techniques were implemented to validate the targets of LHQW. The authors found significant symptoms within the infected group (body weight reduction, and lung/mucosal barrier injury). The intestinal microbiota was similarly perturbed, with observed decreases in *Streptococcus* spp. These symptoms (and viral load) were ameliorated by LHQW. Network pharmacology revealed six putative compounds that may influence the population of microbiota as well as inhibit inflammatory responses *via* Toll-like receptor/nuclear factor kB lung signaling pathways. From the data, the authors inferred that LHQW can be used effectively as a treatment for the symptoms caused by influenza A and the mechanism involved in the regulation of the above-mentioned pathways.

In a clinically focused study, Salomão et al. investigated the role of the placenta in the transmission of chikungunya virus (CHIKV), as the vertical transmission had been reported previously. They studied five placentas of women who were infected with CHIKV during their pregnancy. Findings showed that within 4/5 formalin-fixed placenta samples, there was a positive RT-PCR result for CHIKV. In the 5th case, however, there was a positive RT-PCR in the mother's and baby's serum, validating vertical transmission. Histopathology of the placenta revealed deposits, villus edema, and villous necrosis. Within the placentas that revealed infection, there were increases in CD8^+^ and CD163^+^ cells and proinflammatory (IFN-γ and TNF-a) and anti-inflammatory cells (TGF-B and IL-10) cytokines, relative to non-infected placentas. Additionally, CHIKV was detected in trophoblastic cells and decidual cells. The authors concluded that CHIKV alters the placental histology as a result of perturbing its homeostasis, resulting in a potentially harmful fetal environment.

A study by Singh et al. revealed HupB, a nucleoid-associated protein, to be vital for the survival of *Mycobacterium tuberculosis*, in conjunction with being important to tolerate host-mediated stressors and first-line antibiotics. In their investigation, they revealed that HupB can aid in *M. tuberculosis* survival following macrophage attack, such as *via* nutrient depletion, pH alterations, and oxidative/nitrosative stress. They found a *hupB M. tuberculosis* mutant to be highly susceptible to all the stressors mentioned above. Furthermore, the authors revealed that *M. tuberculosis* can appropriately modulate the expression of HupB in order to mitigate the damage associated with these stressors. The authors also reported that HupB can assist in the generation of resistance to both rifampicin and isoniazid, with the *hupB* mutant illustrating increased susceptibility to both these antibiotics even during a short exposure. When the authors overexpressed HupB, they found *M. tuberculosis* to be significantly more tolerant to macrophage-induced stressors. Interestingly, it was observed that in the mutant devoid of HupB, the permeability of the cell wall was reduced as was the efflux pump expression, which led the authors to suggest that this may play a role in increasing susceptibility to host-mediated stress and antibiotic susceptibility. Lastly, the authors found that when HupB was targeted by a small molecule inhibitor, SD1, *M. tuberculosis* became significantly more susceptible to first-line antibiotics. The authors concluded that HupB may be a potential therapeutic target for combination with first-line antibiotics.

A study performed by Liu et al. aimed to investigate the anti-*Staphylococcus* activity of an organic acid extract of *Portulaca oleracea* (OAPO) *in vitro* and *in vivo*. *S. aureus* is an important clinical pathogen and can be responsible for a variety of infections at different sites in humans and livestock. However, due to the occurrence of methicillin-resistant strains and the introduction of strict laws on antibiotic usage in animals in China, new methods are needed to reduce antibiotic usage to kill S. *aureus*. Using a series of experiments, the authors examined the antibacterial activity of the organic acid of OAPO against *S. aureus*.

*In vitro* antibacterial mechanisms were evaluated based on the integrity and permeability of the cell wall and membrane, scanning electron microscopy, and soluble protein content. A mouse skin wound recovery model was used to assess the antibacterial effects of OAPO on *S. aureus in vivo*. The results showed that the extract of P. oleracea not only inhibits methicillin-resistant *S. aureus* activity *in vitro* but also inhibits *S. aureus*-induced skin damage. Overall, these findings highlight that a botanical extract can inhibit *S aureus in vitro* and *in vivo*, supporting the potential use of OAPO to prevent and control *S. aureus* infections in the near future.

## Author contributions

LL wrote the original draft of the manuscript. GC and CT provided edits to finalize the submission. All authors contributed to the article and approved the submitted version.

